# Paper-based laser-induced graphene for sustainable and flexible microsupercapacitor applications

**DOI:** 10.1007/s00604-022-05610-0

**Published:** 2022-12-30

**Authors:** João Coelho, Ricardo F. Correia, Sara Silvestre, Tomás Pinheiro, Ana C. Marques, M. Rosário P. Correia, Joana Vaz Pinto, Elvira Fortunato, Rodrigo Martins

**Affiliations:** 1 CENIMAT|i3N, Department of Materials Science, School of Science and Technology, NOVA University Lisbon and CEMOP/UNINOVA, Caparica, Portugal; 2 i3N, Department of Physics, University of Aveiro, 3810-193 Aveiro, Portugal

**Keywords:** Laser-induced graphene, Paper electronics, Microsupercapacitors, Sustainable production methods, Flexible devices

## Abstract

Laser-induced graphene (LIG) is as a promising material for flexible microsupercapacitors (MSCs) due to its simple and cost-effective processing. However, LIG-MSC research and production has been centered on non-sustainable polymeric substrates, such as polyimide. In this work, it is presented a cost-effective, reproducible, and robust approach for the preparation of LIG structures via a one-step laser direct writing on chromatography paper. The developed strategy relies on soaking the paper in a 0.1 M sodium tetraborate solution (borax) prior to the laser processing. Borax acts as a fire-retardant agent, thus allowing the laser processing of sensitive substrates that other way would be easily destroyed under the high-energy beam. LIG on paper exhibiting low sheet resistance (30 Ω sq^−1^) and improved electrode/electrolyte interface was obtained by the proposed method. When used as microsupercapacitor electrodes, this laser-induced graphene resulted in specific capacitances of 4.6 mF cm^−2^ (0.015 mA cm^−2^). Furthermore, the devices exhibit excellent cycling stability (> 10,000 cycles at 0.5 mA cm^−2^) and good mechanical properties. By connecting the devices in series and parallel, it was also possible to control the voltage and energy delivered by the system. Thus, paper-based LIG-MSC can be used as energy storage devices for flexible, low-cost, and portable electronics. Additionally, due to their flexible design and architecture, they can be easily adapted to other circuits and applications with different power requirements.

**Graphical Abstract:**

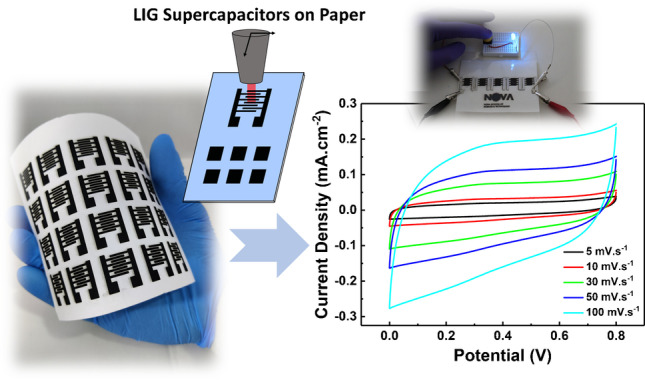

**Supplementary Information:**

The online version contains supplementary material available at 10.1007/s00604-022-05610-0.

## Introduction

The rapid development of portable and wearable technologies has created a great demand for new and improved energy storage and harvesting systems [1–3]. Despite being ubiquitous in modern society, lithium-ion batteries (LIBs) cannot be easily miniaturized and usually rely on harmful electrolytes for functioning [4]. On the other hand, microsupercapacitors (MSCs), specially based on nanomaterials, have been considered promising and viable energy storage systems [2, 3, 5–7]. MSCs are remarkably safe, exhibit high power densities, long life cycles, and fast charge–discharge rates. Moreover, planar two-electrode interdigitated configuration results in small, thin, lightweight, flexible, and easy-to-package devices, in a striking contrast to the conventional sandwich architectures [2, 3, 5, 8]. MSC can also be easily integrated and can function as standalone devices or in tandem with other energy storage units, such as thin-film batteries [4]. So far, MSCs have been fabricated in many flexible substrates, such as plastic, polyimide, fabric, and even paper [2, 9, 10]. In fact, the use of paper in science dates to the nineteenth century, with the creation of the renown litmus paper, which is used as a pH indicator. More recently, the use of paper on flexible electronics has attracted a lot of attention [3, 11, 12]. Paper is easily conformed to a curved surface and compatible with wearable and on-skin technologies [3]. Most importantly, paper is composed of intertwined cellulose fibers; therefore, it is a biocompatible and low-cost substrate, which can be easily recycled or disposed of without long-lasting harmful effects on the environment [13]. MSCs on paper have been fabricated by different approaches, namely stamping [8, 14–16], spraying [17], ink-jet, and 3D printing [18, 19]. However, despite their more than confirmed merits, these techniques may require several production steps and/or exhibit limited pattern configuration and speed [20]. Being a carbon-based material, paper is a suitable candidate for devices fabrication, and can be easily patterned and processed by direct laser writing. This method presents many advantages, namely direct and high precision patternability, simplicity, reproducibility, low waste generation, and speed [21–24]. Upon lasing, the paper fibers are converted to a 3D porous graphene foam, a material referred in the literature as laser-induced graphene (LIG) [13, 25, 26]. Several works have proved that this technique is suitable for the fabrication of high-performance MSCs, mostly in polyimide [27, 28]. Interestingly, it is also possible to atom-dope LIG, thus enhancing or tuning its properties [28, 29]. More recently, Park et al. [30] have shown that paper-based LIG, under different lasing conditions, can present sheet resistances ranging from 61.5 to 9140 Ω sq^−1^. Based on these results, the team fabricated non-volatile read-only memories and resistance-capacitor circuits [30]. Kulyk et al. [26, 31] also showed that it is possible to tune the properties of LIG on paper by varying the lasing parameters (UV laser). They also report LIG exhibiting sheet resistances around 30 Ω sq^−1^, which was then used to manufacture strain, humidity, and temperature sensors. More recently, Pinheiro et al. [13] developed very promising LIG-based biosensors on paper (using a CO_2_ laser). Despite the versatility and many advantages exhibited by LIG, the use of this approach for MSC fabrication on common paper has been seldom reported. Other cellulose-based materials, such as lignin and wood [9, 32–34], have already been tested, but polyimide is still the most widely used substrate for LIG-MSC, which does not represent a viable and sustainable alternative [27, 28, 35].

In this work, we report a low-cost, simple, and one-step approach for the fabrication of in-plane, flexible MSC by direct laser writing on paper. A pre-treatment of the substrate with sodium borate enables the conversion of the cellulose fibers into laser-induced graphene. The resulting devices are free of metallic current collectors and binders and can be designed in any shape. By optimizing the laser parameters (3 W @ 7.6 cm s^−1^), LIG exhibits a sheet resistance as low as 30 Ω sq^−1^, while being highly flexible and resistant towards mechanical deformation. The LIG-based MSC displays a high areal capacity, 4.6 mF cm^−2^, a cycling stability of 96% after 10,000 cycles (0.5 mA cm^−2^), and an energy density of 0.3 μWh cm^−2^ at a power density of 4.5 μW cm^−2^. Finally, the presented methodology allows the fast patterning and adaption to integrated circuits to meet the voltage requirement of different applications.

## Materials and methods

### Paper graphene induction

Whatman chromatography paper grade 1 (Whatman International Ltd., Floram Park, NJ, USA) was immersed for 10 min in a 0.1 M sodium tetraborate decahydrate aqueous solution and left to dry overnight. 0.5 cm × 0.5 cm laser-induced graphene squares were engraved on the treated paper under a 10.6-µm CO_2_ laser system (VLS 3.50, Universal Laser Systems) at powers and speeds ranging from 1 to 5 W and 2.5 to 12.7 cm s^−1^, respectively. The paper substrates were flattened and tapped on a 1.5-mm thick piece of glass prior to engraving. All experiments were conducted at ambient conditions, under nitrogen flow, at a height of 1.8 mm, beam size diameter of 0.127 mm (2.0 lens), and a pulse of 1000 pulses per inch. During this work, ultrapure Milli-Q water laboratory grade (conductivity < 0.1 µS cm^−1^) was used to prepare all solutions. All chemicals were used as purchased.

### LIG characterization

Scanning electron microscopy (SEM) was used to evaluate the morphology of the produced LIG in a Carl Zeiss AURIGA CrossBeam FIB-SEM workstation equipped with an Oxford X-ray Energy Dispersive Spectrometer (Carl Zeiss Microscopy GmbH, Oberkochen, Germany), under high vacuum at an operating voltage of 5 keV. Room temperature micro-Raman measurements were performed using a Horiba Jobin–Yvon HR800 spectrometer equipped with 600 grooves/mm grating, a 532-nm laser line (Ventus-LP-50085, Material Laser Quantum), and a 50 × objective (spot size ≈ 1.3 µm, NA = 0.5). Measurements were conducted with 10-s exposure time, ten accumulations, and a laser power of 1.23 mW. X-ray photoelectron spectroscopy (XPS, Kratos Axis Supra, UK) equipped with a monochromated Al Kα radiation (1486.6 eV). LIG on the paper substrates chemical composition was also studied by EDX by SEM (Hitachi TM 3030Plus Tabletop) to provide a visual inspection of element distribution in the samples. Electrical sheet resistance (Ω sq^−1^) was determined by Hall effect measurements in Van der Pauw geometry in a Biorad HL 5500 equipment at room temperature. These measurements were repeated 3 times for each sample.

### Paper LIG supercapacitors fabrication

The used LIG interdigitated microsupercapacitor electrodes exhibit an areal footprint of 1.8-cm length and 2-cm width as shown in Fig. S1. The fingers’ dimensions were kept at 0.5 cm × 0.08 cm with a fixed interspacing distance of 0.06 cm. All the architectures were designed on Adobe Illustrator and laser engraved onto the borate-treated paper at optimized conditions. The current collectors (0.5 cm × 0.5 cm—Fig. S1) were coated with silver paste and allowed to cure for 1 h at 60 °C. A PVA/H_2_SO_4_ aqueous gel was used as a solid electrolyte. In a normal procedure, 1 g of PVA is dissolved in 10 mL of distilled water at 90 °C under vigorous stirring for 1 h. Then, 0.5 mL of 98% H_2_SO_4_ is added and allowed to mix for another hour. Prior to the electrolyte casting, the MSCs were cured under UV light (NOVASCAN–PSD Pro Series Digital UV Ozone System) to reduce LIG’s hydrophobicity for 15 min at room temperature. The electrolyte is then drop casted onto the devices over an area of ⁓1 cm^2^. The assembled MSCs were allowed to dry overnight at room temperature.

### MSC electrochemical characterization

LIG–MSCs on paper were characterized in a BioLogic SP-50 potentiostat (BioLogic Sciences Instruments) by means of cyclic voltammetry (5 to 10 V s^−1^) and galvanostatic charge discharge (0.015 to 0.5 mA cm^−2^) experiments. Electrochemical impedance spectroscopy (5 mV, 1 MHz to 10 mHz) tests were carried out on a PalmSens 4.0 Potentiostat (PalmSens Compact Electrochemical Interfaces). The MSC specific areal capacitance, *C*_*A*_, was calculated from charge–discharge curves as follows:

1 $${C}_{A}=\frac{I\mathrm{\Delta t}}{A\Delta V}$$

where *I* is the applied current, *∆t* is the discharge time, *A* is the MSC active area, and *∆V* = *V*_2_* − V*_1_ where *V*_2_ is the potential at the beginning of discharge, after the *IR* potential drop, and *V*_1_ is the potential at the end of discharge. Energy (*E*_*A*_) and power (*P*_*A*_) densities per unit area were calculated as follows:

2 $${E}_{A}=\frac{1}{2}\frac{{C}_{A }{\Delta V}^{2}}{3600}$$

3 $${P}_{A}=\frac{{E}_{A }}{\mathrm{\Delta t}}$$

where 3600 is a conversion factor from Ws to Wh. The deformation tests were conducted by mounting LIG-MSC on profiles with radii of 10 mm, 25 mm, and 45 mm followed by CV measurements at 10 mV s^−1^. In a similar way, 1000 bending cycles were applied to the LIG-MSC followed by CV measurements at 100 mV s^−1^.

## Results

### LIG on paper characterization

**Fig. 1:**
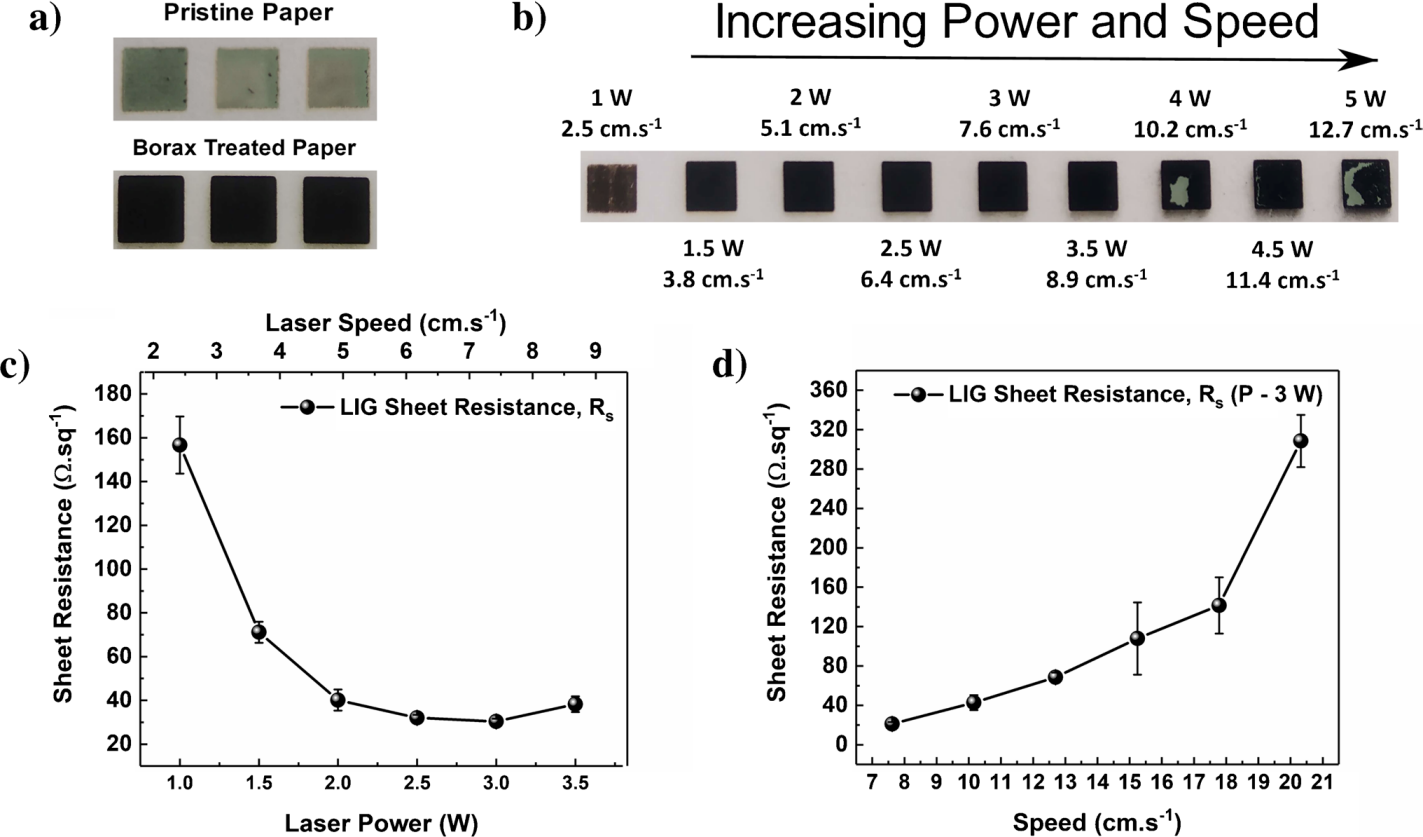
Comparison of lasing effects on pristine paper and borax treated paper (**a**); LIG formation as function of the laser power and speed (**b**) and corresponding sheet resistances (**c**) and LIG films sheet resistance as function of the laser speed at a 3-W power (**d**)

Conventional paper substrates are not the most suitable for graphene direct laser writing. Even at low laser power, a total paper ablation/thermal degradation is likely to occur, as paper is basically composed of cellulose. Laser exposure can heat a substrate above 2000 °C, while cellulose burns around 230 °C [24]. This issue can be circumvented by using inorganic salt additives, such as phosphates and borate derivatives, which increase cellulose thermal resistance [24, 26, 36]. In the present work, Whatman chromatography paper was immersed in a 0.1 M sodium tetraborate (borax) aqueous solution for 15 min and allowed to dry at room temperature. The importance of this first step is shown in Fig. 1a. For the same lasing conditions (1.5 W @ 3.8 cm s^−1^), the borax-treated paper can withstand the photothermal degradation process induced by the laser, leading (in principle) to the formation of graphitic carbon. Without treatment, the substrate is completely vaporized. Up to now, polyimide has been by far the substrate of choice for LIG production. However, the use of fire-retardant additives, in a very simple and elegant way, expands the range of substrates available for LIG processing, such as paper. In a common procedure, it is usual to vary the laser parameters (speed, power, distance, and pulses per inch) to optimize LIG quality. Herein, it was studied the effects of laser power and speed on LIG formation, following a symmetry axis approach, as described by Pinheiro et al. [13]. The distance and pulses per inch were kept constant at the focal point and 1000, respectively. Figure 1b visually depicts the lasing effects on paper. For lower power/speed (1 W @ 2.5 cm s^−1^), the laser barely burns the paper surface. As these parameters are increased, darker samples are obtained, usually reflecting a higher degree of graphene induction. On the other hand, for conditions above 3.5 W @ 8.9 cm s^−1^, the obtained structure is no longer mechanically stable, and it easily tears during LIG formation and/or sample handling.

The lasing parameters’ effects can also be quantified in terms of electrical properties. Figure 1c shows the sheet resistance for LIG produced under the conditions indicated in Fig. 1b. An initial *R*_S_ of 156.71 ± 3.0 Ω sq^−1^ (1 W @ 2.5 cm s^−1^) quickly drops to 30.3 ± 1.3 Ω sq^−1^ for a laser power of 3 W @ 7.6 cm s^−1^. Further increments on laser and speed result in a slight rise on *R*_S_, suggesting that sample deterioration already occurs around 3.5 W @ 8.9 cm s^−1^. Finally, laser power was fixed at 3 W and speed varied from 2.5 to 20.32 cm s^−1^. Again, the speed has a significant effect on LIG electrical properties (Fig. 1d). Below 7.6 cm s^−1^, the substrate was destroyed, and it was not possible to record any measurement. Above this value, the sheet resistance increases significantly, suggesting that for higher speeds there is not enough time or not enough energy is provided to promote efficient LIG formation. Regarding the best obtained sheet resistance, ~ 30 Ω sq^−1^, one may assume that it is quite low for a LIG-based material. A similar result was obtained for optimized LIG on paper treated with a commercial phosphate-based fire retardant [26]. Park et al. [30] reported a sheet resistance of 61.5 Ω sq^−1^ on a commercial fire-retardant paper. LIG *R*_S_ values heavily depend on the substrate and lasing conditions; thus, an accurate comparison across systems can be difficult to establish. To the best knowledge of the authors, the best LIG *R*_S_ are around 10 Ω sq^−1^ for polyimide and cork [37].

**Fig. 2:**
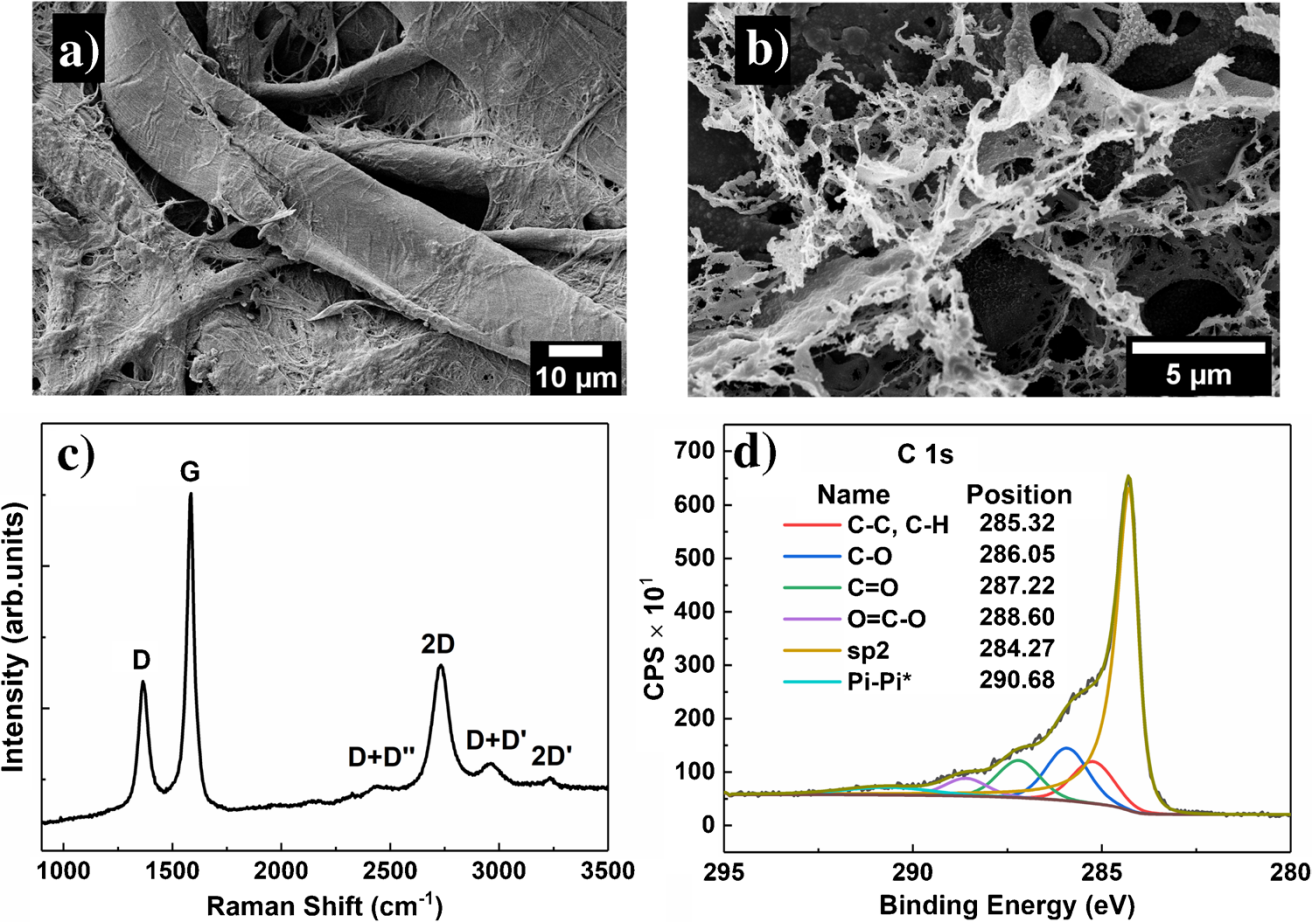
SEM micrographs of paper (**a**) and laser-induced graphene on paper at 3 W @ 7.6 cm s − 1 (**b**). Both micrographs were acquired at 1000 × magnification. Representative Raman (**c**) and XPS (**d**) spectra for the optimized LIG samples

To better understand the type of carbon present in the optimized samples, morphological, structural, and chemical characterization was conducted on LIG prepared at 3 W @ 7.6 cm s^−1^. In morphological terms, paper is composed by cellulose fibers compacted into a substrate exhibiting a random pore distribution (Fig. 2a). Interestingly, upon lasing the substrate at 3 W @ 7.6 cm s^−1^, its morphology is radically changed. The sample seems to open upon laser irradiation, and it is composed of sheets covered in flakes/coral like structures. It is evident that progressive increments on laser power and speed lead to a transformation of the paper substrate, which seems to open from the inside (Supplementary Information—Fig. S4a and Fig. S4b) along with cellulose fiber (Supplementary Information—Fig. S4c) breakdown and formation of open structures composed by sheets and flake-like particles.

From 2 W @ 5.1 cm s ^*−*1^ (Supplementary Information—Fig. S4d) onwards, no significant structural changes can be observed. In fact, these are the conditions at which the *R*_s_ values start to stabilize, reaching its minimum at 3 W @ 7.6 cm s^*−*1^. These results, along with the visual details exhibited in Fig. 1b, suggest that there are critical values of power and speed for LIG formation, above which no further graphenization or material optimization occurs. In fact, above certain lasing conditions, the substrate’s structure can be highly compromised, leading to LIG film tearing and crumbling. Additional information on LIG formation and morphological characterization is provided in the Supplementary Information.

The chemical structure for LIG produced at optimized conditions was studied by Raman spectroscopy (Fig. 2c). The spectrum shows the three characteristic peaks of graphene-derived materials around 1350 cm^*−*1^ (D peak), 1580 cm^*−*1^ (G peak), and 2700 cm^*−*1^ (2D peak) [26, 38, 39]. The D peak is usually associated with the degree of defects in the sample, G peak is generated by graphitic carbon, and 2D peak is related to second-order zone boundary phonons. The presence of the D peak indicates a graphene-defective structure due to distorted sp^2^ carbon networks [40]. For LIG, a single 2D peak around 2700 cm^*−*1^ is a good indication of the presence of graphene [1, 38, 39]. Graphite 2D peak is composed of two peaks (2D1 and 2D2) which are usually observed at 2725 cm^*−*1^ [38, 39]. Moreover, as the intensity ratio *I*_2D/IG_ < 1 (a common observation for LIG), it is assumed that the obtained samples are composed of a few-layer bent graphene sheets rather than single layer [24, 26, 38]. Figure 2d reports the C 1 s spectrum of the laser-induced graphene on paper. The spectrum is dominated by sp^2^ carbon (284.27 eV) along with its corresponding feature π–π* interactions (290.68 eV), which is characteristic of graphene structures. The other spectrum components, namely C–C, C–H (285.32 eV), C–O (286.05 eV), and C = O (287.22 eV), are most likely to arise due to substrate non-processed cellulose and adventitious carbon [10]. The XPS results corroborate the Raman results, showing once again high-quality LIG was produced on paper. Furthermore, the morphological and structural properties of LIG on paper are in good agreement with other reported works, revealing porous structures composed by disorganized few layers of graphene [24, 41].

### MSC fabrication and electrochemical characterization

**Fig. 3:**
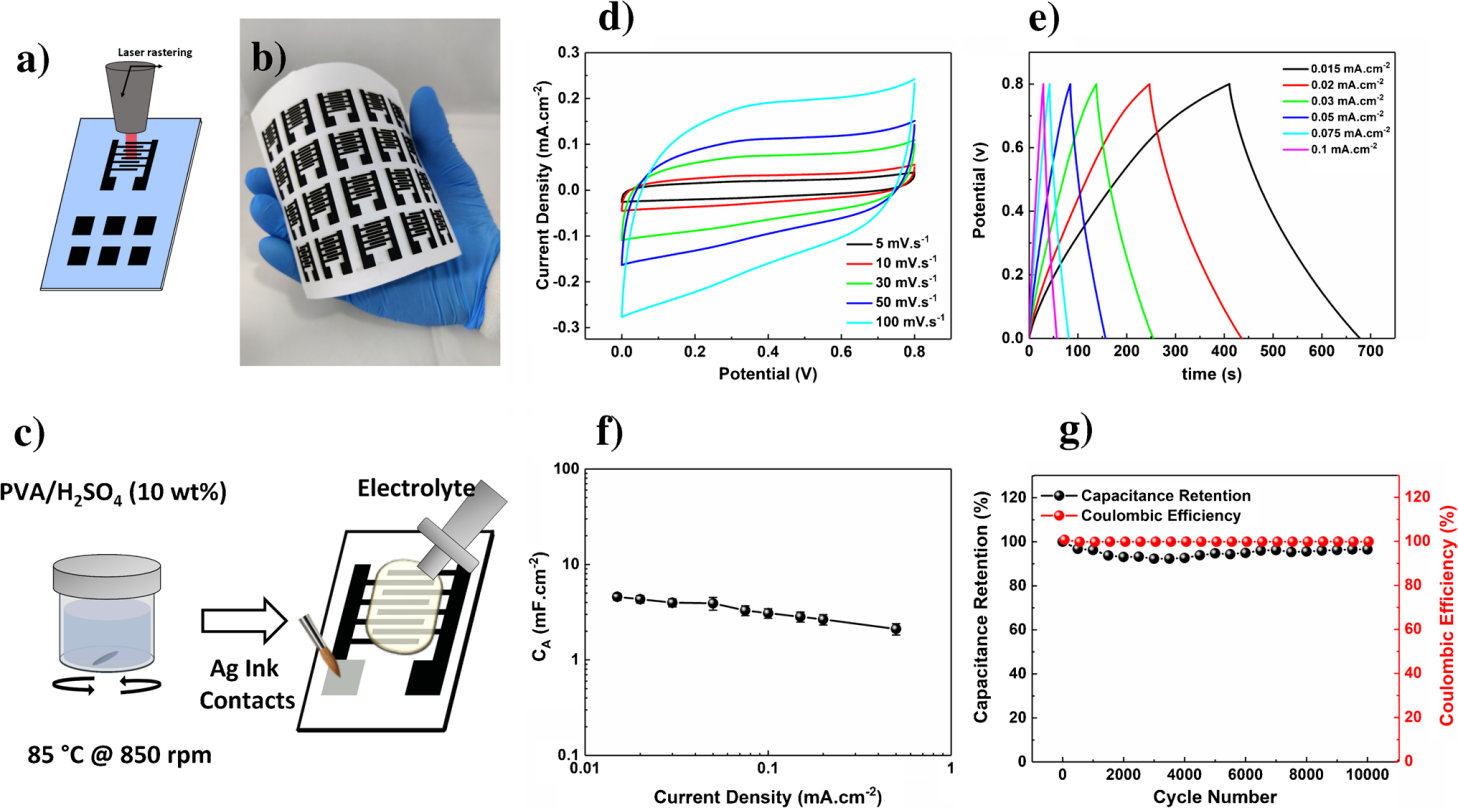
Laser engraving of interdigitated and square patterns on paper (**a**); several and identical interdigitated devices fabricated in a one-step approach (**b**); schematics of LIG-MSC assembly (**c**); CV (d), charge–discharge (**e**) curves, capacitance at different current densities (**f**), and capacitance retention and coulombic efficiency at 0.5 mA cm − 2 (**g**) for LIG-MSC on paper

LIG on paper (3 W @ 7.6 cm s^*−*1^) was used to fabricate interdigitated in-plane MSC. Figure 3a clearly shows that by laser induction, it is possible to fabricate several devices in a one-step, fast, and reproducible way. Their electrochemical and energy storage properties were thoroughly characterized by cyclic voltammetry (CV) and galvanostatic charge discharge (GCD) experiments. For this study, PVA/H_2_SO_4_ was used as electrolyte and LIG worked as both current collector and active material. The CV curves shown in Fig. 3b present a quasi-rectangular shape (5 to 100 mV s^*−*1^), which is characteristic of electric double-layer capacitors (EDLCs) and indicates good electrochemical stability. A similar observation can be made for the GCD curves shown in Fig. 3c. The almost ideal symmetric triangular shapes exhibiting a very low voltage drop is also an indication of a good capacitive behavior. At a current density of 0.015 mA cm^−2^, an areal capacitance (*C*_A_) of 4.6 ± 0.4 mF cm^−2^ was obtained, which is in line with other LIG published works [1, 14]. Even at a current density of 0.5 mA cm^−2^, LIG MSC still exhibits a remarkable *C*_*A*_ of 2.1 ± 2.9 mF cm^−2^ (Fig. 3d). At the same current density, after 10,000 cycles, the devices retain about 85% of their initial capacitance (Fig. 3e). Interestingly, a pronounced capacity loss is observed for the first 2000 cycles, upon which the system stabilizes. The Coulombic efficiency is kept at 99.6% after 10,000 cycles.

**Fig. 4:**
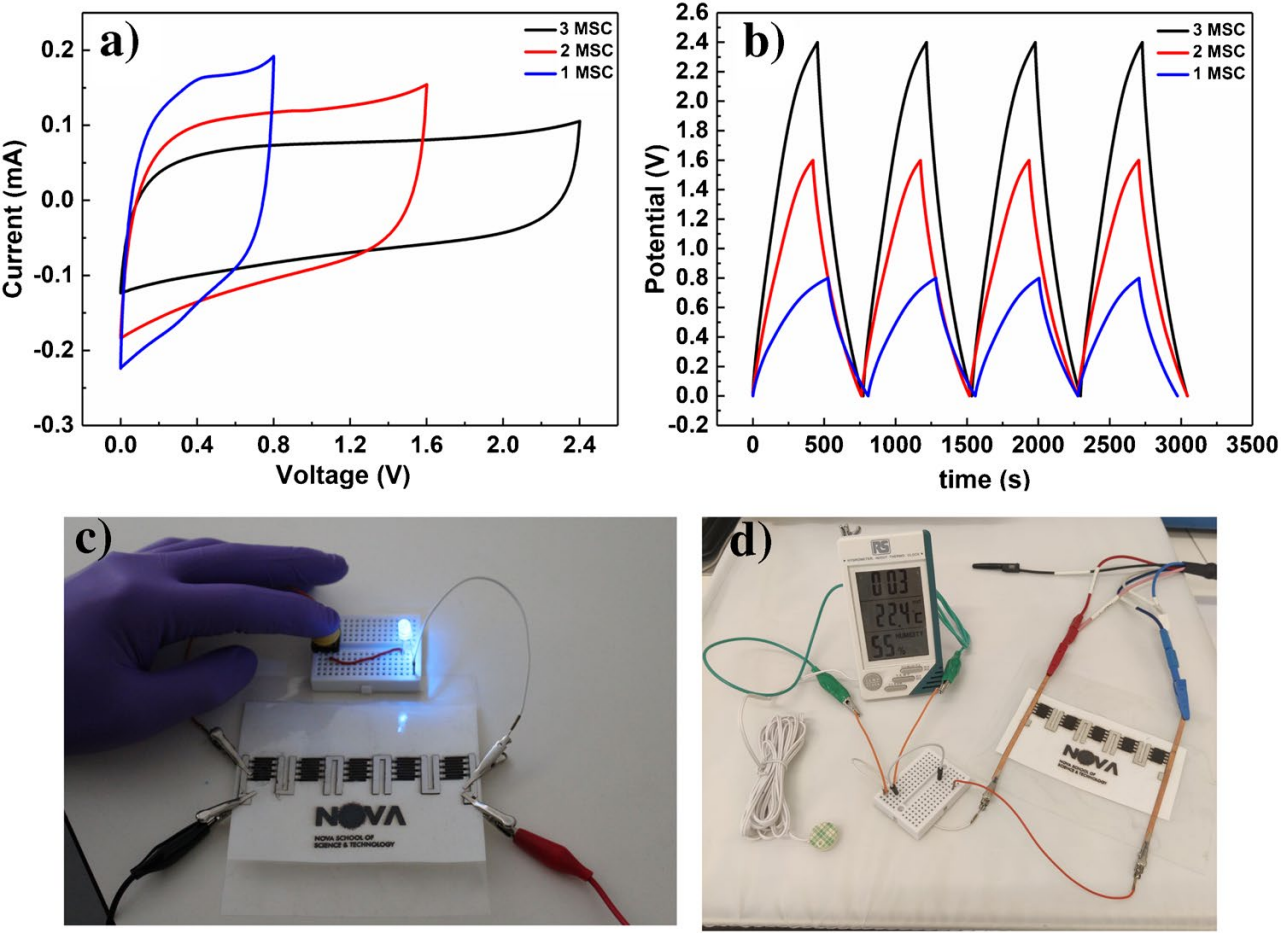
Cyclic voltammograms (**a**) and galvanostatic charge–discharge curves (**b**) for one, two, and three LIG-MSC assembled in series. Led light up (**c**) and humidly sensor (**d**) up by five LIG-MSC in series

To illustrate the scalability and integration of LIG-MSC, flexible devices in series were fabricated and interconnected in a single step on the same paper sheet (Fig. 4). Compared to a single device, the output current on this configuration can be increased by a factor of three, up to 2.4 V, for three devices in series. The GCD curves for the devices in series also exhibit a quasi-triangular shape, with a slight iR drop, as expected for EDLC. Besides its interesting capacitive behavior, LIG-MSCs from paper substrates also exhibit a good control of current densities and working voltage windows (Fig. 4a, b). To further study the LIG-MSC capabilities for flexible applications, five MSC in series were fabricated to open the voltage window up to 4 V and comply with the requirements of a blue LED. The LIG on paper-based power unity was readily available to power the LED for roughly 10 s (Fig. 4c). Additionally, the 5 LIG-MSC in series could as well power a humidity/temperature sensor for roughly 60 s (Fig. 4d). Videos for both applications can be found in the Supplementary Information. These results not only suggest that paper-based LIG-MSC are indeed good candidates for powering flexible and portable electronics, but that they can also be easily adapted to applications with different power requirements. Finally, in terms of energy density, the LIG-MSC exhibit 0.30 μWh cm^−2^ at a power of 4.5 μW cm ^−2^ and 0.12 μWh cm^−2^ at a power of 255.4 μW cm^−2^ (Supplementary Information—Fig. S6). Additional electrochemical analysis and discussion can be found in the Supplementary Information.

Usually, it is not an easy or straightforward task to compare MSC across literature reports due to the different methodologies, shapes, sizes, and amount of material being used. Moreover, the range of metrics obtained for LIG-based MSC is highly dependent on hetero-atom doping, substrate, carbon precursor, among other factors [33, 35]. However, the performance of paper-based LIG-MSC is comparable with several devices assembled by laser carving [9, 14, 35, 42]. In the specific case of paper-based devices, capacitances as high as 22.5 mF cm^−2^ at 300 mV s^−1^ and 21.7 mF cm^−2^ at 0.43 mA cm^−2^ have been reported for stamped MSC using porous graphene inks [8]. Say et al. [43] prepared paper-MSC by spray coating with sheet resistances as low as 100 Ω sq^−1^and specific capacitances around 30 F g^−1^. Zhao et al. [14] obtained similar results top the reported on this work, by developing MSC via a flash foam stamp-inspired fabrication approach. Despite all the merits and advantages of the aforementioned methods, they all depend on multi-step approaches, requiring ink preparation, masks, and/or templates. Huang et al. [44] also used a laser-based method for MSC fabrication on paper, but once again it relied on several steps to obtain a final product. In fact, LIG most attractive features are its simplicity, one-step capabilities, and competitiveness with other more established methodologies. Additionally, the fact that LIG can be doped and combined with other materials to enhance MSC electrochemical performance further opens a wide range of possibilities for LIG-based energy storage devices [35, 45, 46]. In Table 1 are listed some examples of other LIG-based MSC reported on the literature.

**Table 1:** Comparison of LIG-based MSC produced from several sustainable and bio-degradable substrates

Substrate Laser Source	Electrolyte	Capacitance	% Retention	[Ref]
Paper CO_2_ laser	PVA/H_2_SO_4_	4.6 mF cm^−2^ @ 0.015 mA cm^−2^	85% (> 10 000 cycles @ 0.5 mA cm^−2^)	This Work
Cork CO_2_ laser	PVA/H_2_SO_4_	1.35 mF cm^−2^ @ 5 mV s^−1^ 1.43 mF cm^−2^ @ 0.1 mA cm^−2^	106% (> 10 000 cycles @ 0.05 mA cm^−2^)	[47]
Cork/H_3_BO_3_ UV laser	PVA/H_2_SO_4_	4.67 mF cm^−2^ @ 0.1 mA cm^−2^	86% (> 10 000 cycles @ 0.1 mA cm^−2^)	[48]
Lignin CO_2_ laser	PVA/H_2_SO_4_	0.88 mF cm^−2^ @ 10 mV s^−1^	91% (> 10 000 cycles @ 0.02 mA cm^−2^)	[9]
Wood CO_2_ laser	PVA/H_2_SO_4_	1 mF cm^−2^ @ 1 mA cm^−2^	n/a	[49]
Cotton cloth/MnO_2_ Nd:YAG laser	PVA/H_3_PO_4_	54.97 mF cm^−2^ @ 0.5 mA cm^−2^	80.6% (10 000 cycles @ 500 mV s^−1^)	[50]
Fallen leaves Yb fiber laser	PVA/H_2_SO_4_	34.68 mF cm^−2^ @ 5 mV s^−1^	99% after 50 000 @ 100 mV s^−1^)	[51]

For this analysis, it was only considered cellulose-based substrates, such as paper and cork. When compared to pristine cork, lignin, and wood, the induction of graphene on paper leads to better electrochemical properties. However, this effect may be related with the paper porous structure. Upon the lasing process, it is formed a 3D LIG structure with a higher surface area available for the capacitive process. On the other hand, it may explain as well the capacity fading over harsh and repeated mechanical deformations (Fig. S11). The conversion of a high amount of cellulose fibers into LIG deteriorates the paper structure, thus reducing its resistance towards mechanical stress. After 1000 bending cycles at 180°, it is observed a considerable capacitance loss, mostly for the first bending cycles (Fig. S11). This behavior is not compatible with wearable applications, where the devices are expected to be twisted and folded. However, the required encapsulation may help stabilize the devices and render them useful for some flexible applications. Additionally, the results exhibited on Fig. S10 indicate that MSC-LIG can act as conformable energy storage devices. It is also clear from Table 1 that doping LIG results in higher capacitance values, due to pseudocapacitive effects. The use of different type of lasers may potentially influence the properties of induced graphene. For instance, graphene induction by UV lasers is a much more localized effect than for CO_2_ lasers. Therefore, the build-up of conductive pathways may not be as easy. However, multi-lasing approaches easily solve this problem. In fact, the most direct impact of different wavelength lasers is the architecture resolution of the obtained devices.

From Table 1, it seems that doping LIG with pseudocapacitive materials is a good tactic for developing high-performance paper-based devices. Nevertheless, the further development of LIG-MSC on paper is also relies on the optimization of the mechanical stability of the devices. On a broader context, in the implementation of LIG-based devices, some practical aspects must be taken in consideration. First of all, these devices are not expected (or meant) to replace lithography and silicon-based technologies; it is very unlikely that direct laser writing will ever meet the precision and standards required for high-performance product manufacturing. Additionally, LIG usually presents lower figures of merit when compared to graphene powder or other commercially available conductive inks. Nevertheless, it can be of high relevance for simple, cost-effective, fast patterning, flexible systems, which do not need to comply with highly controlled environments and sophisticated fabrication protocols. As for other nanomaterials, LIG is likely poised to occupy a niche in flexible/wearable devices manufacturing, based on a trade-off between production costs and device performance. It is also important to stress the fact that LIG is a cleaner and environmentally friendly production method that may play a role in fighting the ever-increasing amount of electronic waste (e-waste).

## Conclusions

In this work, LIG on paper MSC were successfully fabricated in an interdigitated configuration. The electrodes were prepared by a simple, one-step, sustainable, and cost-effective approach, without requiring the use of metallic current collectors or polymeric binders. Using borax as a fire-retardant agent proved to be crucial for the device’s preparation, as it prevented paper combustion under the laser beam. This strategy allowed to produce LIG on paper, which proved to be an efficient material for microsupercapacitor applications. The obtained MSCs exhibit good electrochemical properties and stability over time, with no significant loss over 10,000 cycles of charge–discharge. In fact, LIG-MSCs on paper have performances comparable to other LIG-based supercapacitors prepared on other substrates such as polyimide. Additionally, the shape, diversity, and ease of interconnecting several devices in series and/or parallel indicate a great potential for the applications of LIG on paper MSC in portable and flexible electronics. However, before exploiting all the LIG on paper capabilities, its mechanical properties must be improved. Nevertheless, the use of biocompatible and biodegradable substrates, such as paper, and clean techniques, as laser carving are promising alternatives for the development of sustainable and greener production methods.

## Supplementary Information

Below is the link to the electronic supplementary material.

Supplementary file1 (PDF 12555 KB)

Supplementary file2 (MP4 1768 KB)

Supplementary file3 (MP4 17979 KB)
