# Supplementary material for: Paper-based laser-induced graphene for sustainable and flexible microsupercapacitor applications
Source: Mikrochim Acta. 2022 Dec 30;190(1):40. doi: 10.1007/s00604-022-05610-0 (PMC9803761; doi:10.1007/s00604-022-05610-0)
Supplement: Supplementary file 1 — Supplementary file1 (PDF 12555 KB) [file 604_2022_5610_MOESM1_ESM.pdf]

Supporting Information

Paper Based Laser Induced Graphene for Sustainable and Flexible Microsupercapacitor Applications

João Coelho[1*], Ricardo F. Correia[1], Sara Silvestre[1], Tomás Pinheiro[1], Ana C. Marques[1], M. Rosário P. Correia[2], Joana Vaz Pinto[1], Elvira Fortunato[1], and Rodrigo Martins[1*]

[1]CENIMAT—i3N, Department of Materials Science, School of Science and Technology, NOVA University Lisbon and CEMOP/UNINOVA, Caparica, Portugal

[2]i3N, Department of Physics, University of Aveiro, 3810-193 Aveiro, Portugal

***E-mail** = jcm.coelho@fct.unl.pt; rfpm@fct.unl.pt

MSC Design

Fig. S1 depicts the structures and dimensions of the studied MSC. The fingers length and width were kept at 0.5 cm x 0.08 cm, respectively with a finger inter-distance of 0.06 cm.

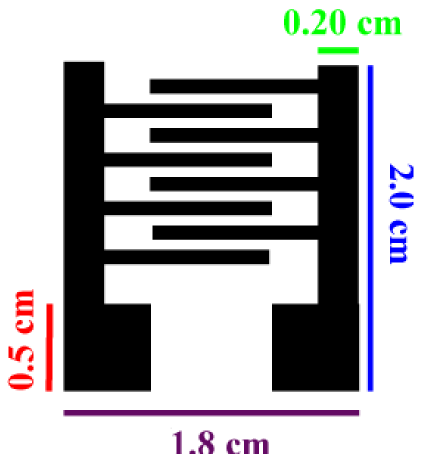

Figure S1: LIG-MSC design and dimensions.

LIG Formation and Atomic Composition

It is widely accepted that during the lasing process on paper treated with a borate fire-retardant, the additive decomposes through endothermic processes (heat sink action) and releases water, thus enhancing paper resistance towards photothermal degradation and avoiding ignition[1]. Moreover, the borylation of paper, also catalyzes oxygen removal and cellulose dehydration, which enables the production of graphene in a one step process, in milliseconds through the overlapping of multiple lasing pulses[2, 3]. These effects can be visually verified by EDX mapping (Fig. S2a and S2b). In a quantitative way, the oxygen content considerably drops upon LIG formation, from 48% on paper to 10.3% on LIG prepared at 3 W @ cm.s^{-1} (Fig. S2c and S2d). Again, as verified for R_S, it seems that the bulk conversion of cellulose to LIG is obtained around 2 W @ 5.1 cm.s^{-1} (Fig. S3).

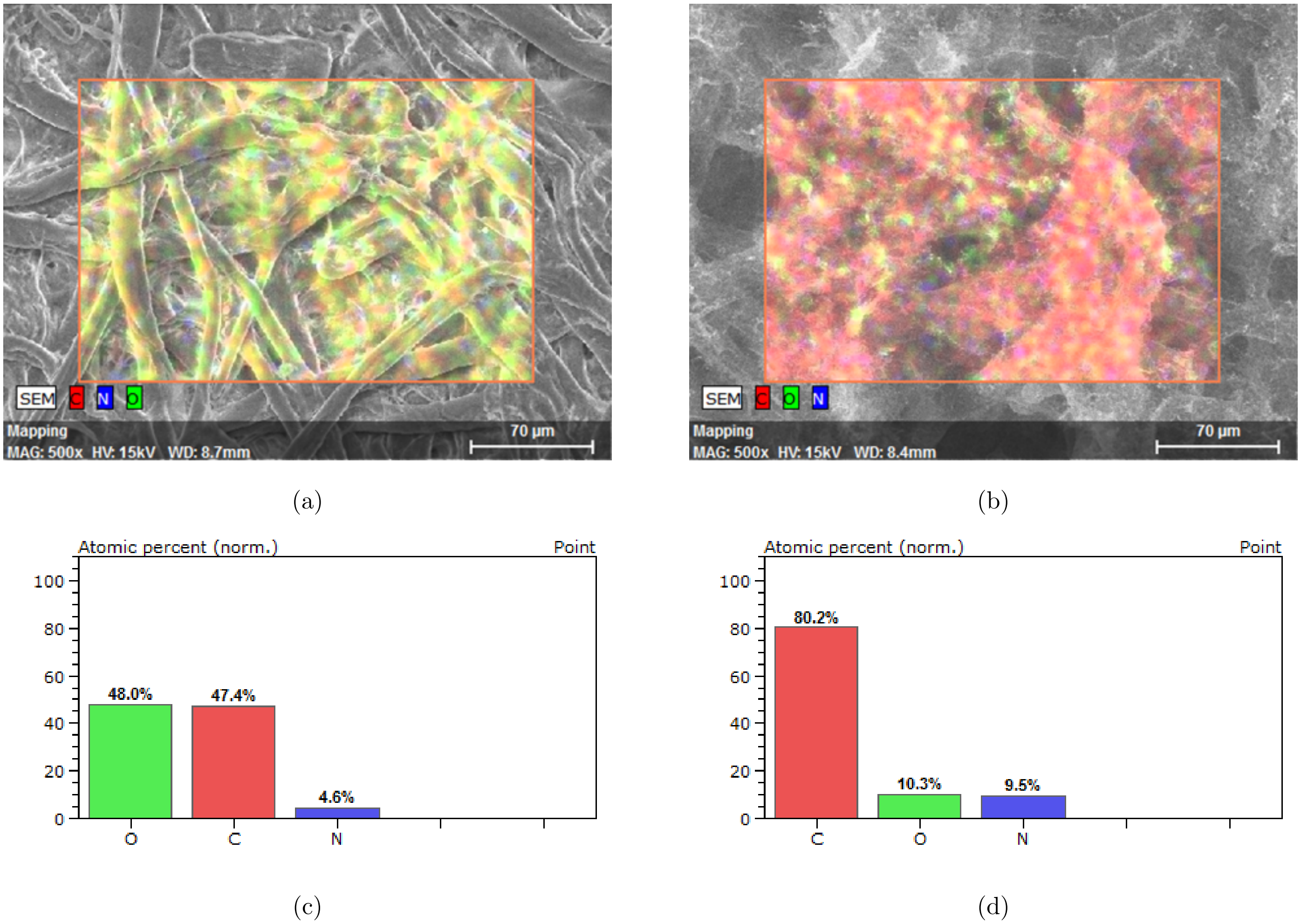

Figure S2: EDX mapping and corresponding atomic percentages of pristine paper (a and c) and optimized LIG (b and d).

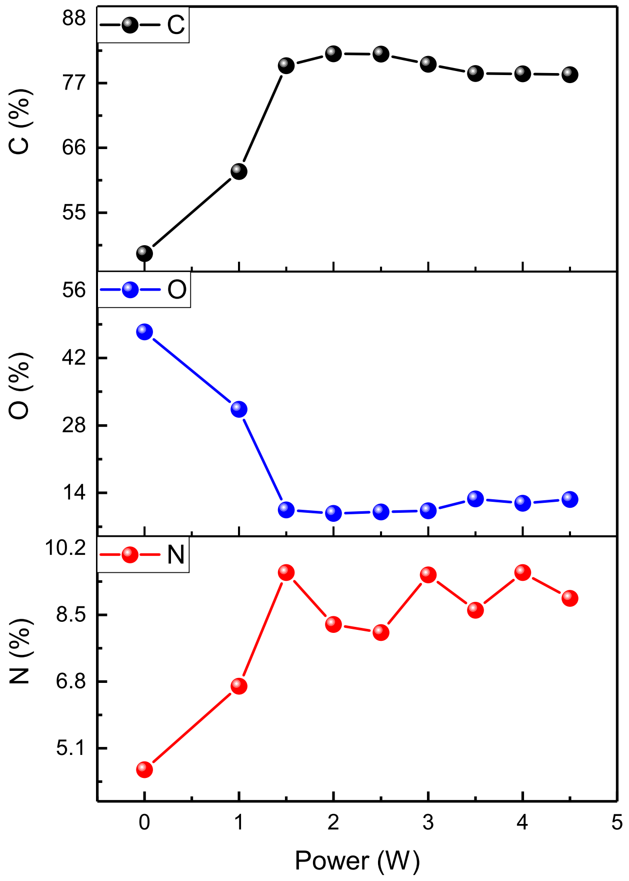

Figure S3: LIG atomic composition as function of the laser power.

It is important to point out the fact that a cellulose-based substrate is quite inhomogeneous and LIG local properties may slightly vary from the average on the whole sample. Additionally, as LIG production is based on a multiple lasing approach it is possible that graphene quality is lower towards the edge of the patterns. Therefore, LIG composition can vary across the sample. Nevertheless, the obtained atomic compositions, as an overall measurement rather than local, points to the successful production of LIG.

LIG Morphological Characterization

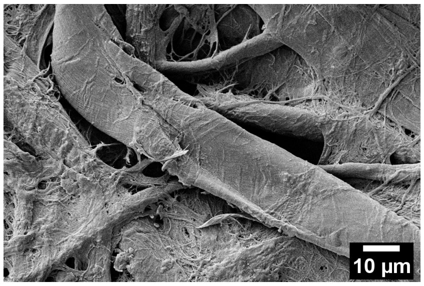

(a) Paper

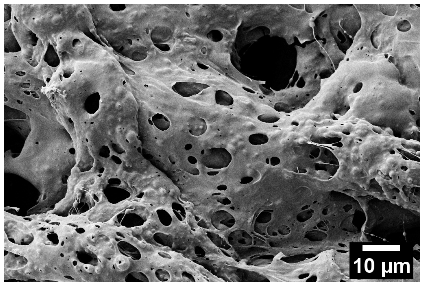

(b) 1.0 W @ 2.5 $cm.s^{-2}$

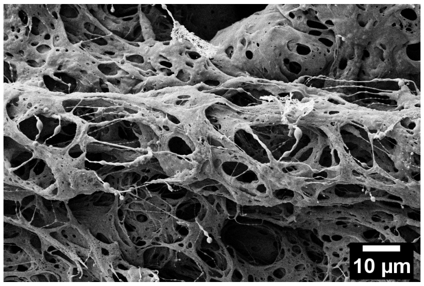

(c) 1.5 W @ 3.8 $cm.s^{-2}$

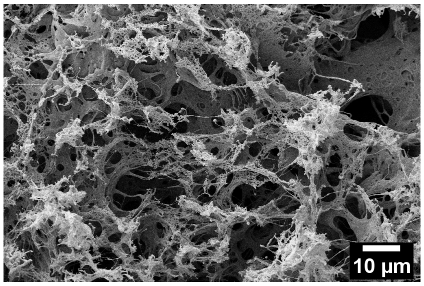

(d) 2.0 W @ 5.1 $cm.s^{-2}$

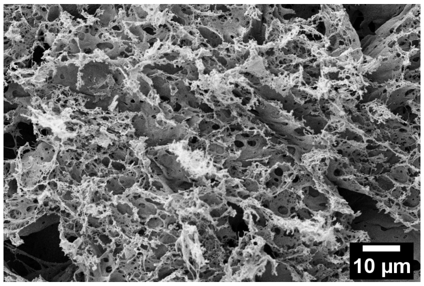

(e) 2.5 W @ 6.4 $cm.s^{-2}$

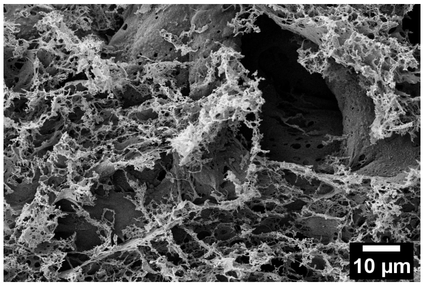

(f) 3.0 W @ 7.6 $cm.s^{-2}$

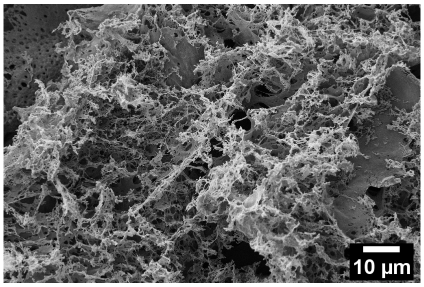

(g) 3.5 W @ 8.9 $cm.s^{-2}$

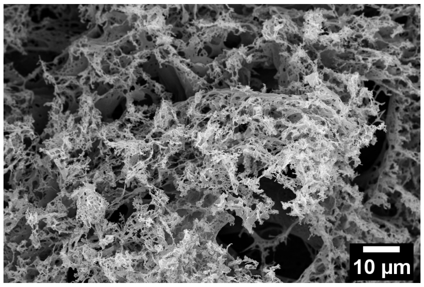

(h) 4.0 W @ 10.2 $cm.s^{-2}$

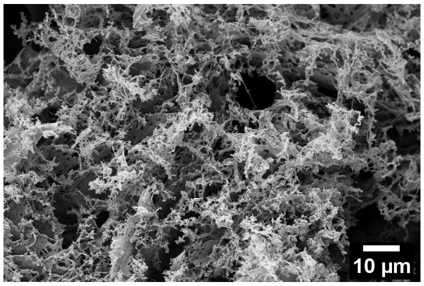

(i) 4.5 W @ 11.4 $cm.s^{-2}$

Figure S4: SEM micrographs (a-i) revealing the effects of incremental laser power and speed on the process of graphene induction on paper. All micrographs were acquired at a 1000x magnification.

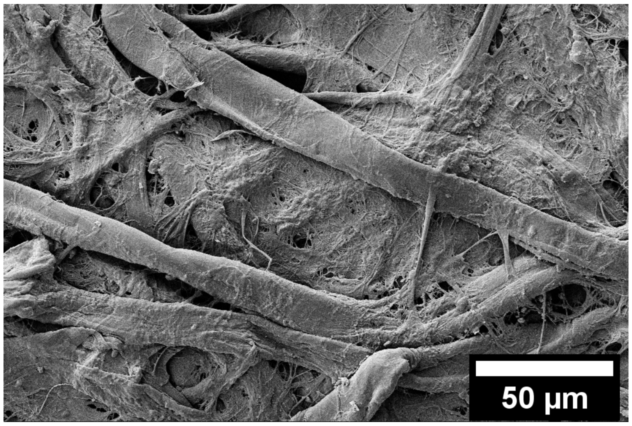

(a) Paper @ 500x

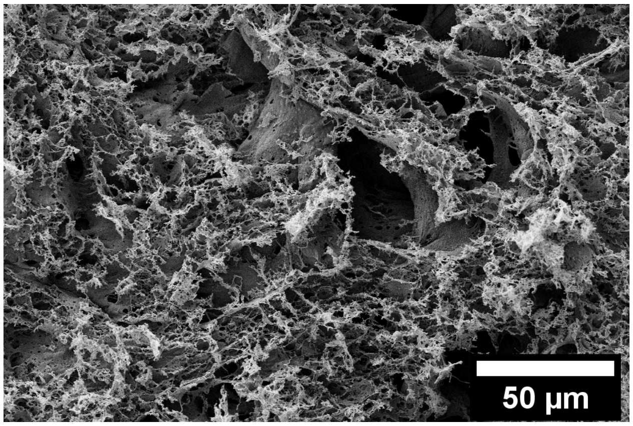

(b) LIG @ 500x

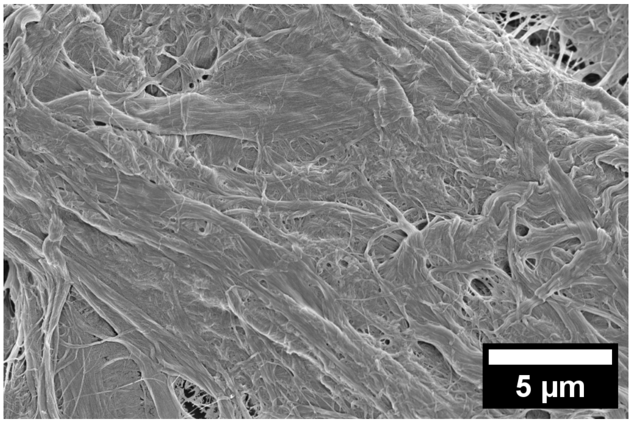

(c) Paper @ 5000x

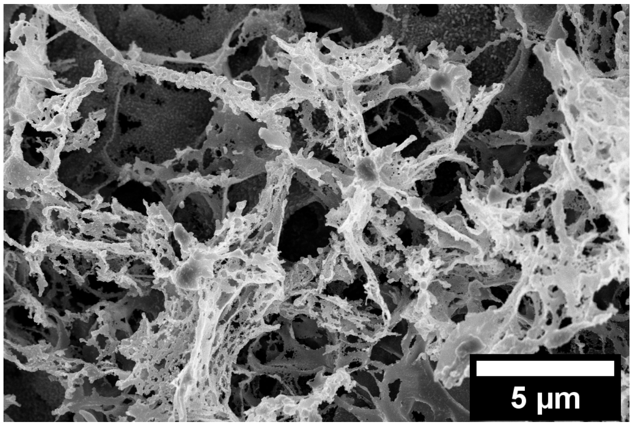

(d) LIG @ 5000x

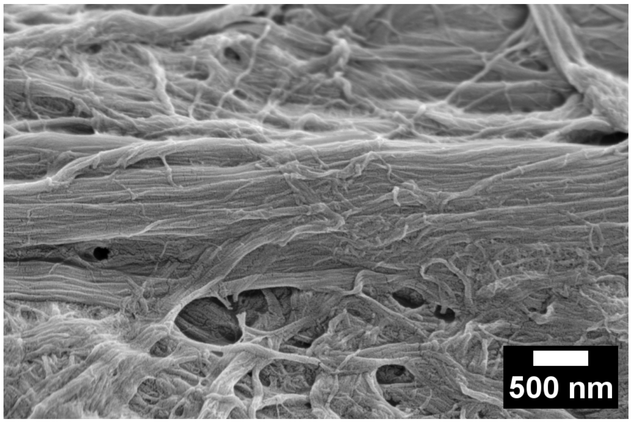

(e) Paper @ 20000x

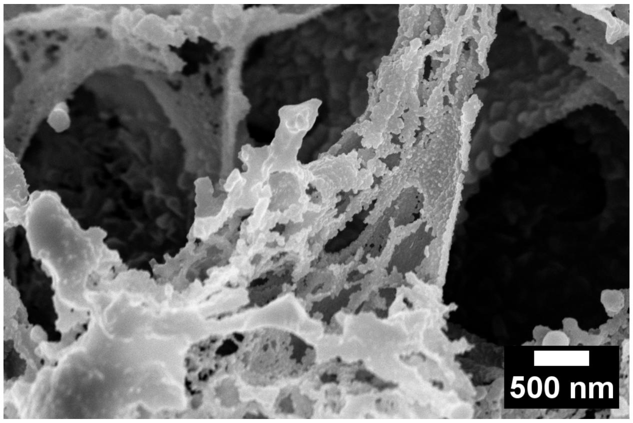

(f) LIG @ 20000x

Figure S5: SEM micrographs of pristine paper and LIG obtained at different magnifications. Figure (f) clearly reveals LIG open structure covered by sheets of flakescoral like graphene.

Further Electrochemical and Mechanical Characterization

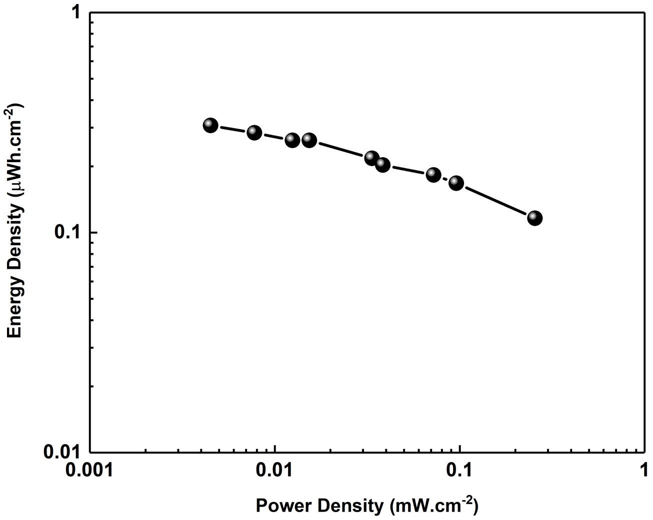

Figure S6: LIG-MSC Ragone plot.

Fig. S7 shows LIG-MSC CV and charge-discharge curves for higher scan rates and current densities, respectively. In Fig. S7a is observed a loss of capacitive behavior towards higher scan rates, as the square box shape, typical of EDLC, is no longer observed above 500 mV/s. These results are consistent with the charge-discharge experiments which suggest a limiting current density of 0.5 mA/cm^2.

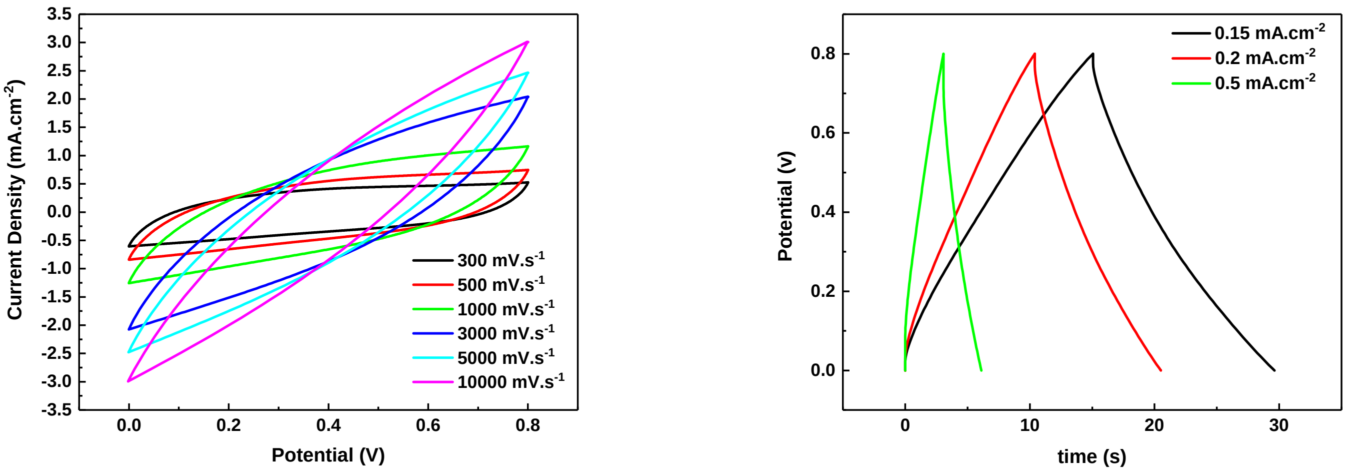

(a) (b)

Figure S7: LIG-MSC CV curves from 300 mV/s to 10000 mV/s (a) and charge-discharge curves up to 0.5 mA/cm^2 (b).

EIS analysis (Fig. S8) revelas a equivalent series resistor (ESR) of 120 Ω which is relatively low for this type of device. More interestingly, a negligible charge-transfer resistance indicates a good electrochemical

performance of the LIG-MSC.

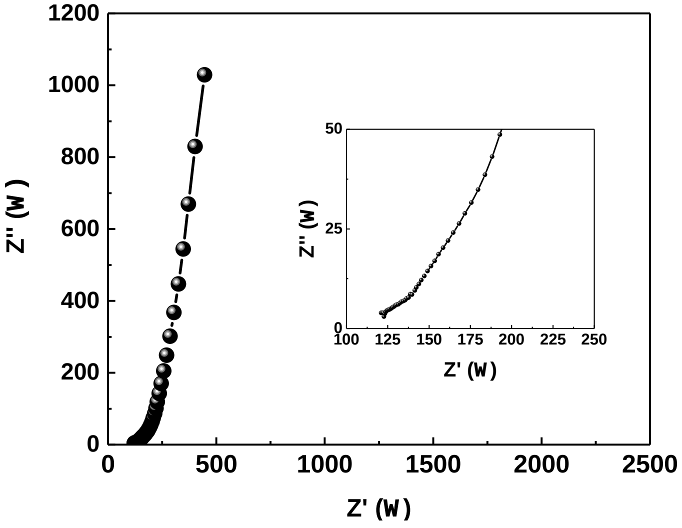

Figure S8: LIG-MSC Nyquist plot.

In Fig. S9 are depicted the cyclic voltammograms at 100 mV.s^{-2} and galvanostatic charge-discharge curves (0.3 mA.cm^{-2}) for LIG-MSC devices assembled in parallel. As it is expected for this type of configuration, the CV currents increase with the number of devices. In this case, the specific capacitance for three devices in parallel is as high as 7.45 mFcm^{-2}. A similar pattern is observed for the charge-discharge experiments, where the measured discharge time for three devices in parallel is roughly three times larger than for a single device. These results further confirm the capability of adapting the LIG-MSC towards applications with different power and energy requirements.

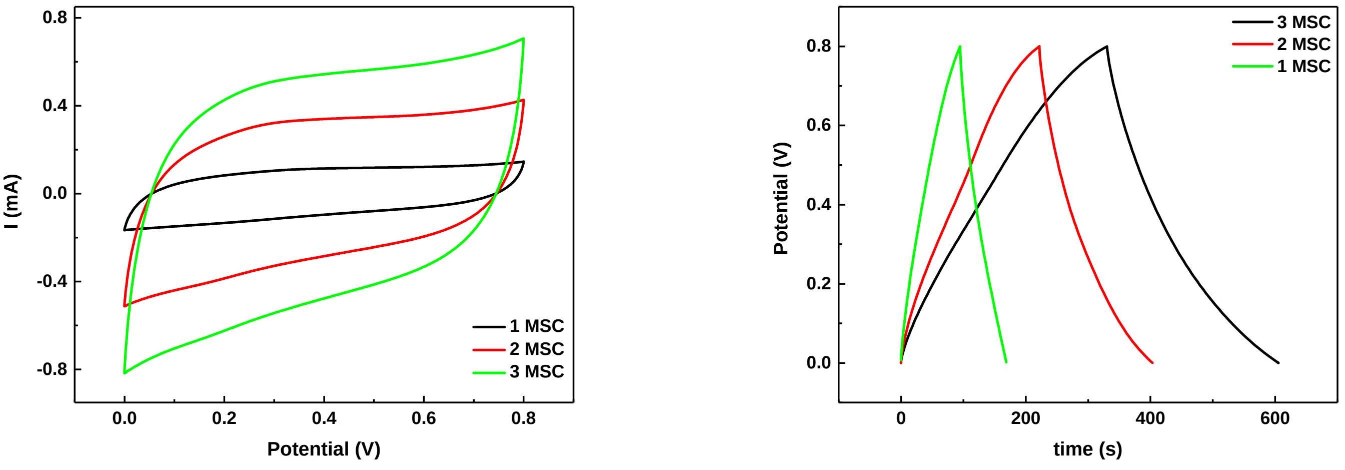

(a) (b)

Figure S9: CV (a) and Galvanostatic charge-discharge (b) curves for LIG-MSC assembled in parallel.

Besides electrochemical performance, the manufactured devices should be capable of withstanding mechanical deformation. Thus, the devices were subjected to bending deformation tests as shown in Fig. S10a-c.

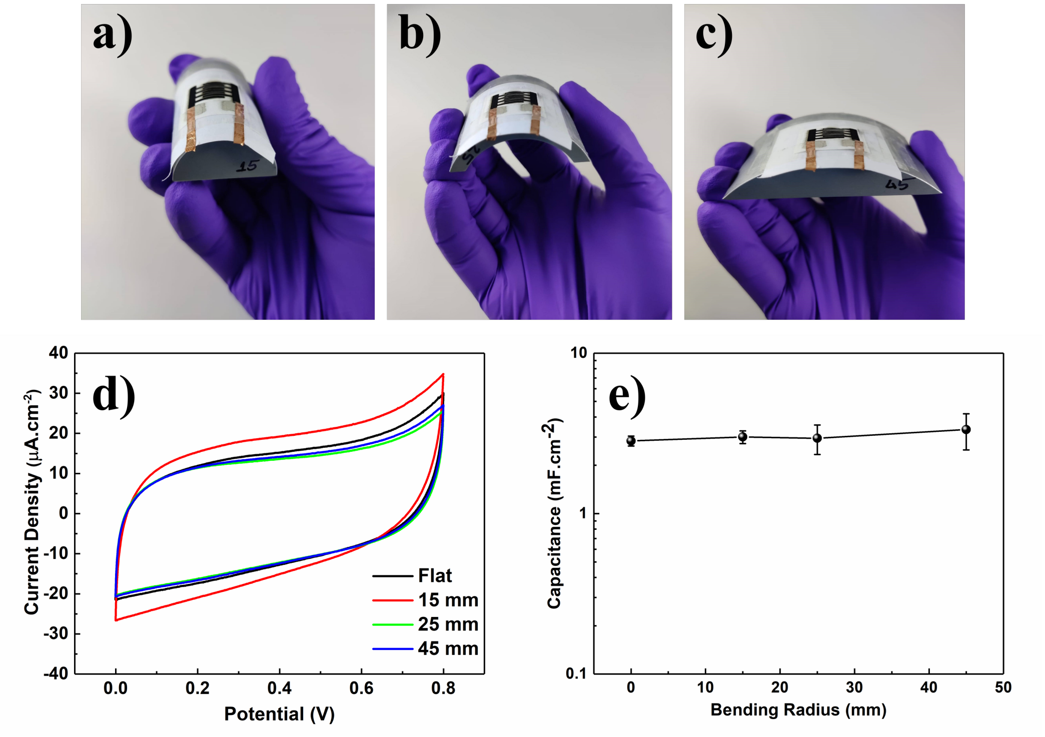

Figure S10: LIG-MSC under mechanical deformation of 10 mm (a), 25 mm (b) and 45 mm (c); CV curves of MSCs tested under different bending deformations (d) and capacitance retention (e) at the different bending angles. The presented data results from cyclic voltammetry experiments conducted at a scan rate of 5 mV.s^{-1}

When compared with the flat device, the cyclic voltammogram curves keep their typical shape (Fig. S10d). This effect is of high relevance as it indicates that the MSC electrochemical performance is not significantly affected by mechanical deformations, thus indicating a good flexibility for future incorporation with wearable electronics, such as sensors or electronic skin. Additionally, the MSC exhibits negligible capacitance degradation at higher bending radius (Fig. S10e). It is clear that the produced in-plane supercapacitors can withstand mechanical deformations without sacrificing their electrochemical performance. This fact can be explained by the good contact of LIG with the solid electrolyte. After drying, the electrolyte keeps the active material in place, thus avoiding LIG peeling off, which could lead to capacitance loss. However, under continuous mechanical stress the device's capacitance significantly decreases upon harsh one thousand bending cycles at 180 degrees (Fig. S11). However, the tests were conducted under heavy mechanical deformation, which may not replicate real-life applications. In fact, to be used the device or platform should be protected and encapsulated to withstand for instance washing cycles, which in turn will stabilize the devices. These results along with

the good cycling stability once again demonstrate LIG-MSC planar supercapacitors capabilities as flexible, or surface conformable energy storage devices.

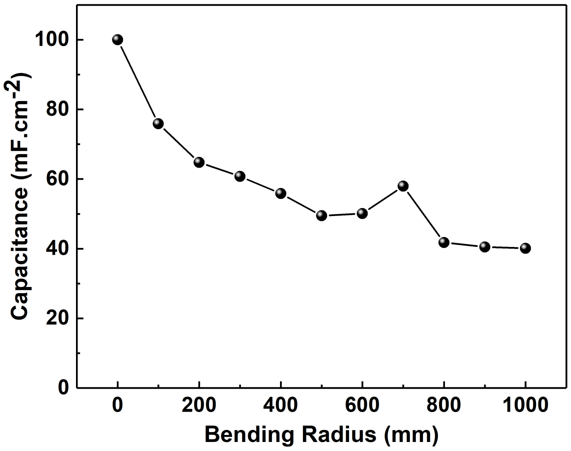

Figure S11: LIG-MSC capacitance fade upon 180° cycles. The capacitance was obtained ay 100 $mV.s^{-1}$.

Supplementary References

[1] Mehmet Dogan, Sengul Dilem Dogan, Lemiye Atabek Savas, Gulsah Ozcelik, and Umit Tayfun. Flame retardant effect of boron compounds in polymeric materials. *Composites Part B: Engineering*, 222:109088, 2021.

[2] Yieu Chyan, Joseph Cohen, Winston Wang, Chenhao Zhang, and James M Tour. Graphene art. *ACS Applied Nano Materials*, 2(5):3007–3011, 2019.

[3] Yieu Chyan, Ruquan Ye, Yilun Li, Swatantra Pratap Singh, Christopher J Arnusch, and James M Tour. Laser-induced graphene by multiple lasing: toward electronics on cloth, paper, and food. *ACS nano*, 12(3):2176–2183, 2018.
